# Loss of E-cadherin disrupts ovarian epithelial inclusion cyst formation and collective cell movement in ovarian cancer cells

**DOI:** 10.18632/oncotarget.6588

**Published:** 2015-12-13

**Authors:** Pui-Wah Choi, Junzheng Yang, Shu-Kay Ng, Colleen Feltmate, Michael G. Muto, Kathleen Hasselblatt, Kyle Lafferty-Whyte, Lellean JeBailey, Laura MacConaill, William R. Welch, Wing-Ping Fong, Ross S. Berkowitz, Shu-Wing Ng

**Affiliations:** ^1^ School of Life Sciences, The Chinese University of Hong Kong, Hong Kong, China; ^2^ Department of Obstetrics/Gynecology and Reproductive Biology, Brigham and Women's Hospital, Boston, Massachusetts, USA; ^3^ School of Medicine, Griffith University, Meadowbrook, Australia; ^4^ GeneGo, Thomson Reuters, New York, New York, USA; ^5^ Center for Cancer Genome Discovery, Dana-Farber Cancer Institute, Boston, Massachusetts, USA; ^6^ Department of Pathology, Brigham and Women's Hospital, Boston, Massachusetts, USA

**Keywords:** ovarian cancer, three-dimensional culture, inclusion cyst, tumor invasion, collective movement

## Abstract

Increased inclusion cyst formation in the ovary is associated with ovarian cancer development. We employed *in vitro* three-dimensional (3D) organotypic models formed by normal human ovarian surface epithelial (OSE) cells and ovarian cancer cells to study the morphologies of normal and cancerous ovarian cortical inclusion cysts and the molecular changes during their transitions into stromal microenvironment. When compared with normal cysts that expressed tenascin, the cancerous cysts expressed high levels of laminin V and demonstrated polarized structures in Matrigel; and the cancer cells migrated collectively when the cyst structures were positioned in a stromal-like collagen I matrix. The molecular markers identified in the *in vitro* 3D models were verified in clinical samples. Network analysis of gene expression of the 3D structures indicates concurrent downregulation of transforming growth factor beta pathway genes and high levels of E-cadherin and microRNA200 (miR200) expression in the cancerous cysts and the migrating cancer cells. Transient silencing of E-cadherin expression in ovarian cancer cells disrupted cyst structures and inhibited collective cell migration. Taken together, our studies employing 3D models have shown that E-cadherin is crucial for ovarian inclusion cyst formation and collective cancer cell migration.

## INTRODUCTION

Ovarian cancer is the leading cause of death among gynecologic diseases in Western countries [1]. Because the disease is diagnosed mostly in advanced stage and there is a lack of effective therapy to treat the frequent relapses, patients with malignant epithelial ovarian tumors have a poor 5-year survival rate of about 30% [2]. Although recent genomic sequencing endeavors and prior studies have found specific gene mutations frequently found in different ovarian tumor types [3-5], the mechanisms of tumor initiation and progression remain elusive.

Many epidemiologic studies have shown that low numbers of pregnancy and infertility, hence high ovulation numbers, are linked to increased incidence of ovarian cancer [6, 7]. High ovulation numbers are also associated with increased invaginations of ovarian surface epithelial lining in forms of inclusion cysts [8]. Historically, Müllerian metaplasia of ovarian surface epithelial (OSE) cells located in the cortical inclusion cysts has been suggested as one pathologic change for the development of ovarian cancer [9, 10] and occasional cases of ovarian intraepithelial neoplasia are identified histologically in the inclusion cysts or on the surface epithelia [9, 11]. Although recent studies have attributed the development of high-grade serous ovarian carcinoma with a p53 signature to the epithelial cells in distal fallopian tube [12, 13], induction of ovarian surface epithelial transformation was observed in several mouse models for ovarian tumors [14-17], and p53 mutations were also found in nonserous tumors such as high-grade endometrioid cancer [15, 16], suggesting potential involvement of the OSE cells and inclusion cysts in ovarian tumor development.

**Figure 1:**
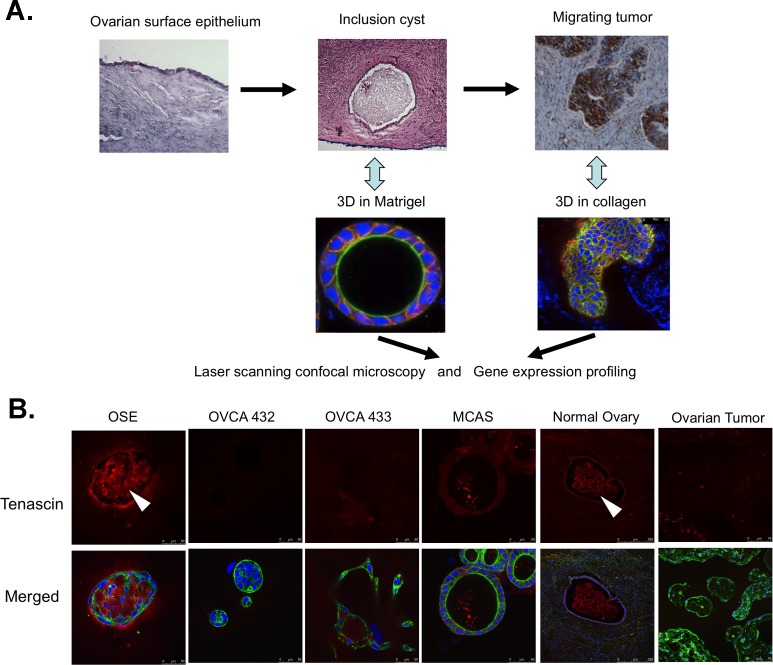
Employment of three-dimensional *in vitro* cultures to study *in vivo* cellular structures in clinical samples **A.** A schematic illustration of sequential employment of 3D Matrigel cultures and 3D collagen I cultures to study clinical inclusion cyst and migration tumors. **B.** Immunostaining of tenascin (red) in the 3D Matrigel cultures of different cell lines and normal ovaries and ovarian tumors. The deposition of tenascin inside the spheroids and inclusion cysts are indicated by arrowheads. The merged images are the overlaid images of tenascin (red), F-actin (green) and nucleus (blue).

*In vitro* three-dimensional (3D) cultures are increasingly employed to study organogenesis [18] and morphogenesis of cellular structures in normal and disease states [19-21]. Gene expression and genomic analyses of cells growing in 3D cultures have shown that the models can be used to depict characteristics of clinical tumor progression and metastasis and the 3D gene profiles exhibit high degree of similarity to those of primary tumor profiles [22, 23]. We are interested in studying the molecular events in association with morphologic changes of ovarian inclusion cysts formed by normal and transformed ovarian cells. In this study, we employed two different matrices to produce 3D cultures with morphologies resembling ovarian inclusion cysts and migrating tumors (Figure 1A). Network analysis of the expression profiles has identified a key pathway for the inclusion cyst formation and migration of ovarian cancer cells. The genes involved in the pathway can be verified in the clinical samples, suggesting that the *in vitro* 3D models can be employed to recapitulate and manipulate morphologic changes that occur *in vivo* and probe the mechanisms involved in pathologic diseases.

## RESULTS

### Different morphologies of 3D structures formed by ovarian epithelial cells in Matrigel

Figure 1A illustrates the design of 3D cultures for studying the morphologic and molecular changes resembling the *in vivo* cellular structures in clinical samples. Single cells derived from normal human ovarian surface epithelial (OSE) primary cultures and HPVE6E7-immortalized OSE cell lines, as well as a panel of ovarian cancer cell lines formed 3D cellular structures after 7 to 14 days in Matrigel, which contained the majority of basement membrane proteins (Figure S1). The majority of the 3D structures were spheroids with variable sizes. The spheroids formed by OSE cells had diameters of around 100 to 150 μm and contained lumens. Compared with the cancer spheroids, the OSE spheroids showed low compactness and simple organization. The spheroids formed by cancer cell lines had a size of about 50 to 100 μm with two different lumen structures: MCAS, SKOV3 and RMG1 had distinct lumens with a diameter > 20 μm, whereas the spheroids formed by CAOV3, OVCA420, OVCA432, OVCA3 and TOV112D cell lines did not have any lumens, or were very small if one was present. The serous ovarian cancer cell line OVCA433 was distinctly different in that it formed only tubular branch-like 3D structure. As a reference, tumor-associated fibroblasts (TAFs) formed amorphous clusters of cells, which were distinctly different from the spheroids formed by ovarian epithelial cells.

### Elevated expression of epithelial and polarity biomarkers in the cancer spheroids and similar expression patterns between *in vitro* Matrigel models and clinical samples

The 3D structures of OSE7, MCAS, OVCA432, and OVCA433, representing four distinct morphologies in Matrigel, were selected for detailed immunofluorescence staining (Figure S2). All the spheroids (OSE7, MCAS and OVCA432) showed positive staining of collagen IV, a marker of the basal membrane [24]. They differed, however, in the pattern of epithelial and cell polarity markers such as the adherens junction markers β-catenin and E-cadherin, and the apical surface marker GM130. The normal OSE spheroids were weak in both β-catenin and E-cadherin staining, and showed random staining of GM130. In contrast, both the MCAS and OVCA432 cancer spheroids showed intense β-catenin and E-cadherin staining as well as a strong GM130 staining in the apical surface, indicating that the cancer spheroids were polarized. Similar polarized spheroid organization was also observed for the spheroids of other cancer cell lines (data not shown). Supplementary Videos S1-2 show z-stack scanning images across the spheroids formed by OSE7 and MCAS stained with E-cadherin to exemplify the differences between ovarian cancer cell lines and OSEs. For the branching structures formed by OVCA433 cells, there was good β-catenin and collagen IV staining, but very weak E-cadherin and no localized GM130 staining. The clusters formed by TAF in the Matrigel did not show any positive expression of E-cadherin (data not shown).

To determine if the *in vitro* Matrigel models are clinically relevant, we compared the expression patterns of several extracellular matrix proteins and epithelial markers in the Matrigel 3D cultures with those of clinical samples. As shown in Figure 1B, the extracellular matrix protein tenascin was highly expressed in the OSE spheroids. In some spheroids, the overexpressed protein was deposited in the lumens of the spheroids. Similar expression pattern was found in the archived normal ovarian cysts, with tenascin found along the cyst epithelia and in the lumens. In contrast, the 3D structures formed by the ovarian cancer cells did not show intense staining of tenascin, neither was there specific staining in the ovarian tumors. The OSE spheroids stained weaker than OVCA432 and MCAS spheroids for the basement membrane protein laminin V (Figure S3A). The OVCA433 ovarian cancer 3D structures had very faint laminin V staining. As for the clinical samples, the normal ovarian surface epithelia and inclusion cysts showed much weaker staining of laminin V when compared with the staining of laminin V in serous ovarian tumor tissues. In concordance with the intense staining of E-cadherin in MCAS and OVCA432 Matrigel spheroids (Figure S2B), serous ovarian tumor tissues had more intense E-cadherin staining than normal ovarian surface epithelia and inclusion cysts (Figure S3B). Overall, we saw similar expression and localization patterns of epithelial markers between the *in vitro* 3D Matrigel models and clinical samples.

### Collective movement demonstrated by ovarian cancer cells during migration from the cyst-like structures

**Figure 2:**
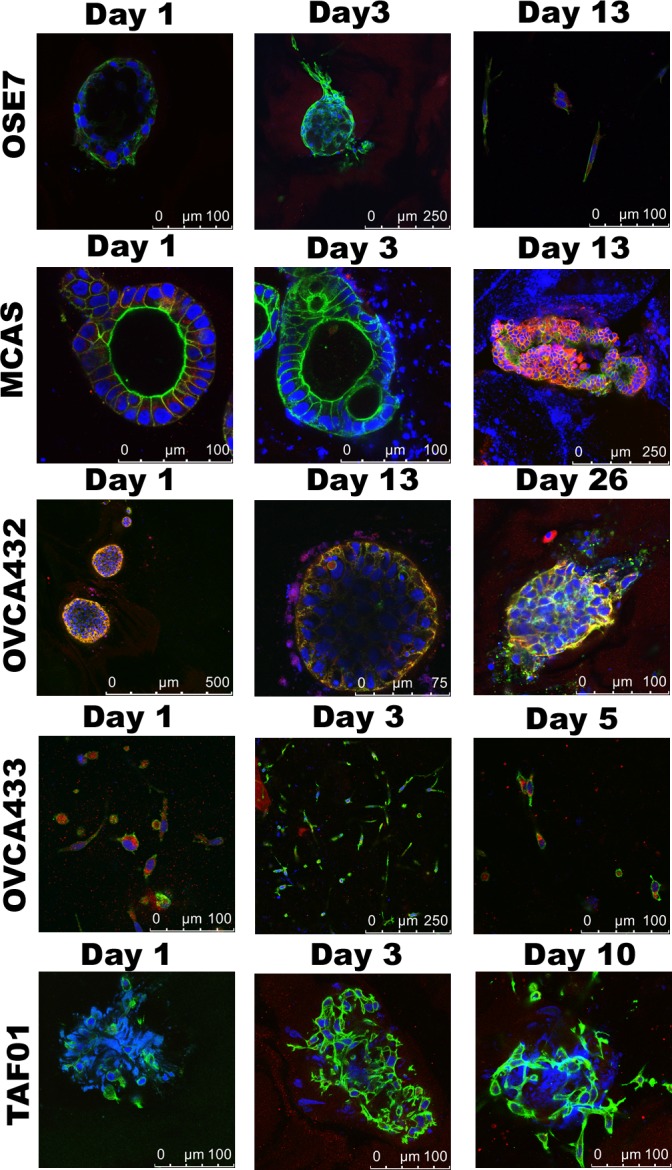
Cell migration in 3D Collagen I cultures Confocal images of the collagen I 3D cultures for OSE7, MCAS, OVCA432, OVCA433, and TAF cells taken on different days. Shown are merged figures of E-cadherin (red), F-actin (green), and nucleus (blue) staining.

The 3D Matrigel structures of OSE7, MCAS, OVCA432, and OVCA433 were transferred to Collagen I matrix and observed for 7 to 20 days (Figure 2). The OSE7 spheroids remained as single spheroids. There were some single cells branching out from the spheroids beginning on Day3. The expression of E-cadherin was lower than that of the cancer spheroids. Spheroids formed by MCAS cells were seen migrating to each other and merged. Cells were emerged from the spheroids as early as Day 3. However, unlike OSE spheroids, z-stack scanning images showed the emerging cells as a cohesive multicellular mass from the spheroids, suggesting that the cancer cells were migrating collectively. This can be appreciated in Supplementary Video S3. E-cadherin level was first down and then elevated and maintained at a high level when the cells migrated out. The merging of spheroids and collective movement of OVCA432 cancer cells was similar to MCAS cancer cells, albeit it took more time to develop. Another difference between the collagen I 3D structures of these two cancer cell lines is that F-actin was distinct from the E-cadherin staining in the MCAS structures, whereas F-actin fluorescence overlapped completely with E-cadherin in the OVCA432 3D structures. In addition, a small number of single cells were found detached from the OVCA432 mass in the later period of migration. The E-cadherin expression throughout the growing period was maintained at a high level. Both the videos of the 3D collagen structures for MCAS (Supplementary Video S3) and OVCA432 (Supplementary Video S4) suggest the collective movement and elevated expression of E-cadherin in the migrating cancer cells. For the OVCA433 3D structure, the branching network expanded with little changes in E-cadherin expression. Tumor-associated fibroblasts maintained the same structure as in Matrigel throughout 10 days in Collagen I, with little E-cadherin staining.

### Gene expression profiling identified a miRNA-mediated E-cadherin/TGFβ switch as the major pathway in association with inclusion cyst formation and cancer cell movement

**Figure 3:**
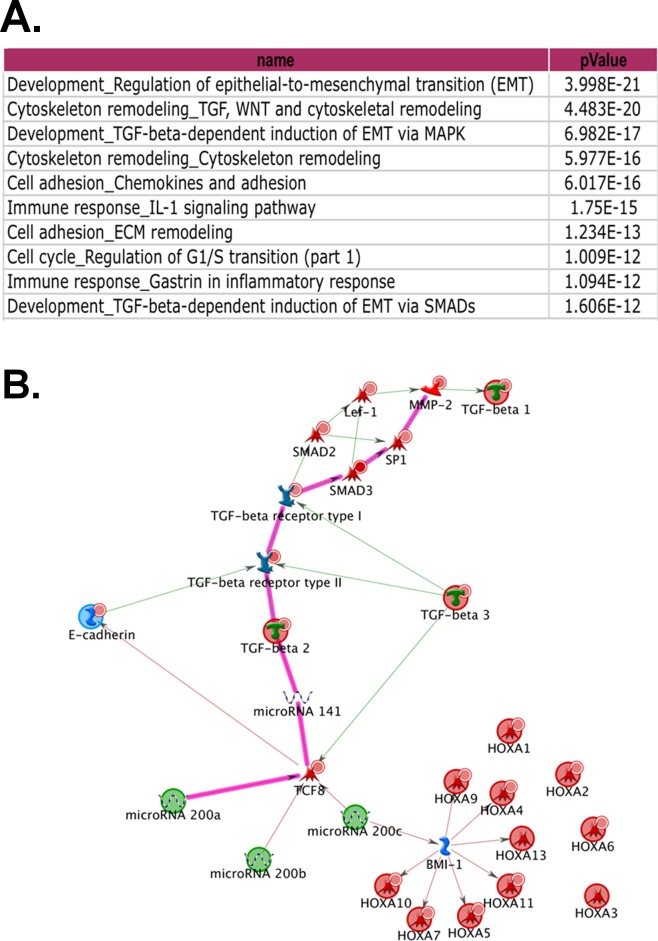
Network analysis of the gene expression profiles of monolayer cells and 3D cultures **A.** The highly significant signaling pathways identified with MetaCore™ Suite. **B.** The key network route generated by MapEditor™ module of the MetaCore™ program, with the most significant one highlighted in pink.

To uncover the underlying key pathways for inclusion cyst formation and ovarian cancer cell migration, we carried out gene expression profiling of two normal OSE cell lines (OSE7 and OSE9), MCAS, OVCA432, and OVCA433 growing under three conditions (as monolayer, in 3D Matrigel, and in 3D Collagen I matrix). The top five ranking signaling networks identified after the analysis of normalized expression data involve transforming growth factor beta (TGFβ), epithelial-to-mesenchymal transition (EMT), and cytoskeleton remodeling (Figure 3A). A network route was mapped based on canonical pathways and expression data to show the suppression of transforming growth factor beta II (TGFβ2) pathway by microRNA200 (miR200) family (Figure 3B, block route). MiR200 family is known to downregulate both the TGFβ2 pathway and a homeobox transcription repressor, TCF8/ZEB1, a repressor of E-cadherin expression [25, 26]. Hence, inclusion cyst formation and ovarian cancer cell movement in the 3D structures are likely the result of an imbalance between the opposing E-cadherin and TGFβ functions. Supplementary Table 1 highlights the expression patterns of the representative genes in this network in the cell lines under the three culture conditions. Compared to the monolayer cultures, OVCA432 and MCAS Matrigel cultures showed elevated levels of E-cadherin expression and diminished expression of ZEB1, whereas OSE cells and OVCA433 cells showed increased expression of MMP2 and reduced levels of TGFβ2. In the collagen 3D cultures, both OVCA432 and MCAS cells did not maintain high levels of E-cadherin expression as in the Matrigel cultures, but they still showed low level of ZEB1 expression. Compared with these two cancer cell lines, OSE and OVCA433 cells maintained high levels of MMP2 and ZEB1 in both Matrigel and collagen 3D cultures. It is intriguing that the gene expression of OVCA433 3D structure was intermediate between OSEs and the other two cancer cell lines. Together with OVCA432 and MCAS, the three cancer cell lines showed essentially much lower TGFβ2 expression compared with the OSE cells. However, OVCA433 had significantly elevated ZEB1 level similar to those of the OSE structures, and correspondingly, significantly subdued expression of E-cadherin.

**Figure 4:**
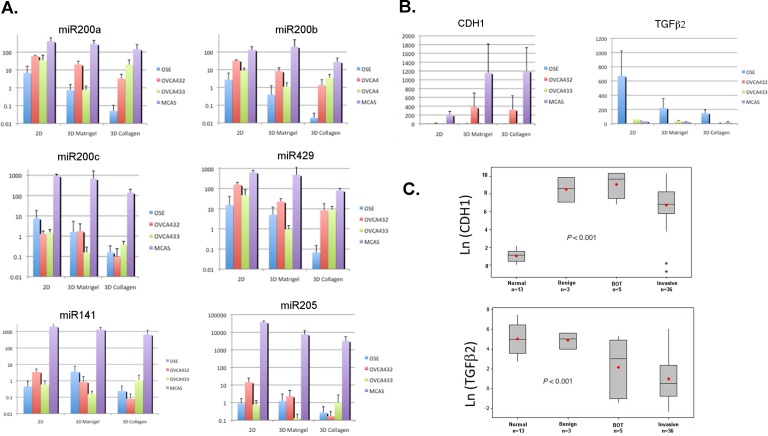
Quantitative real-time PCR to validate the microarray data and evaluate the miR200 expression in monolayer and 3D cell cultures **A.** qRT-PCR results of miR200 family members in the monolayer (2D) and 3D cultures. **B.** qRT-PCR results of E-cadherin (CDH1) and TGFβ2 in the monolayer (2D) and 3D cultures. **C.** qRT-PCR results of E-cadherin (CDH1) and TGFβ2 in the clinical samples including normal ovaries, benign tumors, borderline (BOT) tumors, and invasive ovarian carcinomas.

We performed qRT-PCR to verify the microarray gene expression data and to evaluate the miR200 levels in the monolayer and 3D structures of different cell lines. As shown in Figure 4A, the levels of miR200a, miR200b, and miR429 showed very similar expression patterns. The levels in OSE cells decreased from monolayer to 3D cultures, whereas the levels in all three cancer cell lines remained significantly higher than those of OSE cells in the collagen 3D structures. In contrast to these three miRNAs, the patterns shown by miR200c, miR141, and to a lesser extent by miR205, are obviously different. Except for the MCAS cell line that maintained high levels of these miRNAs, the levels in the structures formed by OVCA432 and OVCA433 were not very different from those formed by OSE cells. In Figure 4B, qRT-PCR showed that the E-cadherin levels in MCAS and OVCA432 cells were much higher than those in OSE and OVCA433 cells in the 3D culture conditions. For TGFβ2, the levels in all cell lines were down-regulated gradually from monolayer to the 3D structures. However, the levels in OSE cells remained elevated relative to all of the three cancer cell lines. We also investigated the expression of E-cadherin and TGFβ2 in epithelial cells microdissected from frozen ovarian tissues (Figure 4C). E-cadherin mRNA levels in benign, borderline and invasive ovarian tumor cells were significantly higher than the levels in ovarian surface epithelial cells collected from normal ovaries (*P* < 0.001). In contrast, the levels of TGFβ2 mRNA in both borderline and invasive tumor cells were significantly lower than the levels in normal ovarian surface epithelial cells and benign tumors (*P* < 0.001). Hence, beside protein biomarkers, RNA expression patterns from the *in vitro* 3D models can also be verified in the clinical samples.

### Knockdown of E-cadherin expression in ovarian cancer cells disrupted cyst formation and inhibited collective cell migration

**Figure 5:**
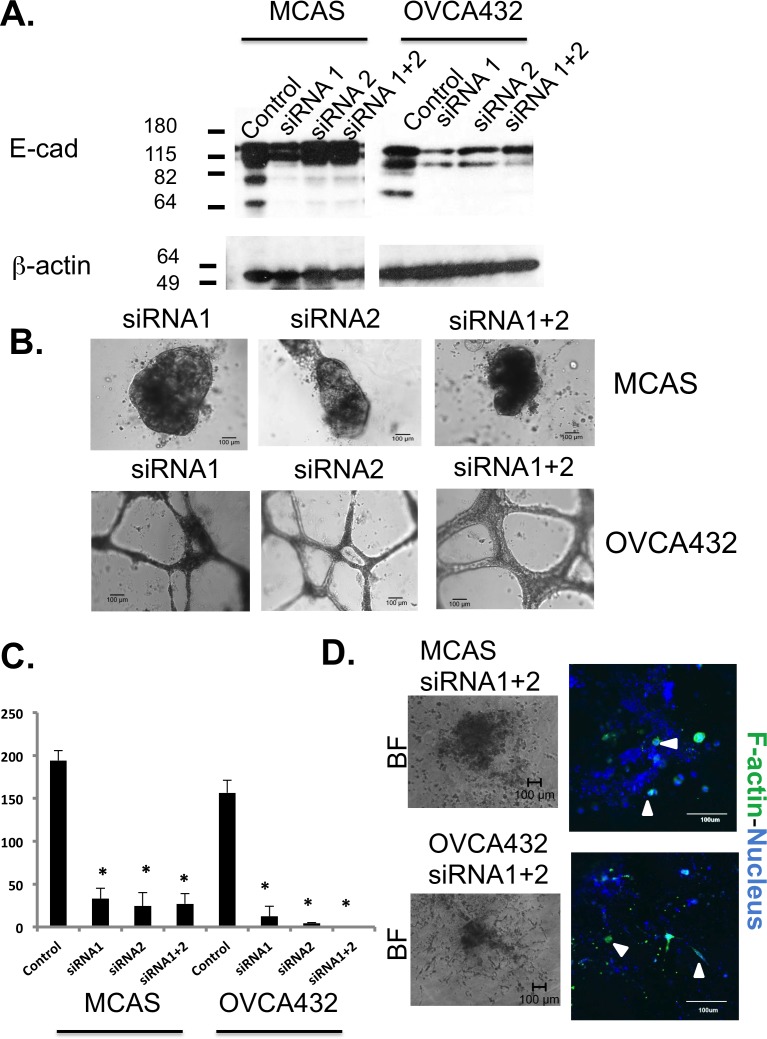
Knockdown of E-cadherin expression in MCAS and OVCA432 cells significantly changed spheroid formation and tumor cell migration **A.** Western blot analysis to show the expression level of E-cadherin in MCAS and OVCA432 cells after control and knockdown siRNA transfections. β-actin was used as the internal control for protein loading. **B.** Bright field images of MCAS and OVCA432 cells after knockdown siRNA transfections and cultured in Matrigel. Scale bar represents 100 μm. **C.** Number of spheroids formed by MCAS and OVCA432 cells after control and knockdown siRNA transfections and cultured in Matrigel. Equal number of single cells was used in the 3D Matrigel cultures for all the cell lines. The results represent mean ± standard deviation of two independent experiments. *, *P* < 0.05. **D.** Bright field and confocal images of MCAS and OVCA432 spheroids after knockdown siRNA transfections and grown in the 3D Collagen I culture for 26 days. Green and blue colors represent cytoskeleton stained by phalloidin and nucleus stained by Sytox Green, respectively. The scale bar is 100 μm. Arrowheads indicate the single cells emerging from the deformed spheroids.

As high E-cadherin level was found in both 3D Matrigel and Collagen I cancer spheroids, we performed a small interfering RNA (siRNA) knockdown experiment to investigate the role of E-cadherin in cyst formation and cell migration in ovarian cancer cell lines MCAS and OVCA432. After transfection with the knockdown siRNAs, Western blot analysis showed that E-cadherin expression was only marginally reduced in MCAS cells, whereas the expression was significantly reduced in OVCA432 cells (Figure 5A). Surprisingly, MCAS cells transfected with the knockdown siRNAs formed larger spheroids (diameter >400 μm) than MCAS cells transfected with control siRNA in Matrigel (Figure 5B). Instead of spheroid formation, OVCA432 cells transfected with knockdown siRNAs formed connecting web-like structures in Matrigel. The numbers of spheroids were significantly lowered for both cancer cell lines transfected with knockdown siRNAs (Figure 5C). Lastly, knockdown of E-cadherin also inhibited the collective movement of MCAS and OVCA432 cells after transfer to Collagen I matrix. The 3D structures of both MCAS and OVCA432 cells were deformed and single cells emerged from the structures after 26 days of culture in Collagen I matrix (Figure 5D).

## DISCUSSION

In this work, we have employed two 3D culture models sequentially to study the morphologic and molecular changes during inclusion cyst formation and the exit of cancer cells from the cyst structures. Morphologic analysis showed that the expression patterns of the epithelial and extracellular matrix markers of the *in vitro* culture models closely resemble the expression patterns in the clinical specimens (Figures 1 and 2 and Figures S2 and S3). Immunofluorescence showed overexpression and deposit of extracellular matrix protein tenascin in the lumen of the OSE 3D spheroids, similar to what was seen in clinical ovarian inclusion cysts (Figure 1B). Tenascin has been reported to be involved in parenchymal-mesenchymal interactions during morphogenesis and wound healing and plays a role in regulating the ovarian cycle [27]. On the other end, laminin V and epithelial marker E-cadherin were expressed highly in both ovarian cancer spheroids and tumor tissues (Figure S3). In conjunction with the similarities between the 3D models and clinical samples in the expression patterns of E-cadherin and TGFβ2 mRNA revealed by RT-PCR (Figure 4B and 4C), current data support the notion that the *in vitro* 3D cultures may recapitulate cellular and molecular events of the *in vivo* structures.

One hallmark of malignant cancer is the capability of cancer cells to invade local tissues and metastasize to distant sites [28]. Our collagen 3D culture models for MCAS and OVCA432 ovarian cancer cells showed induced migration and exit of cancer cells from the spheroid structures (Figure 2 and Supplementary videos S3-4). The migrating cancer cells were interconnected as a cohesive mass with elevated expression of E-cadherin, suggesting that the cancer cells were migrating in a collective manner as described for tissue remodeling and in some cancer invasion studies [29-31]. The dominant conceptual framework over the past 20 years for tumor cell dissemination has been epithelial-mesenchymal transition (EMT). The EMT framework suggests that transformed epithelial cells migrate as individual elongated mesenchymal-like single cells [32]. However, emerging evidence has supported the involvement of the less well-understood collective migration mechanisms displayed in the invasion of many solid tumors [29, 33, 34]. Collective cell migration is characterized as cells being physically and functionally connected such that the integrity of cell-cell junctions is maintained [29]. Our network analysis of gene expression profiles has identified concurrent elevated expression of E-cadherin and downregulation of TGFβ2 expression in ovarian cancer 3D spheroids and clinical samples (Figure 3), suggesting that ovarian cancer 3D cultures and clinical tumors are more epithelial-like than OSE 3D cultures and normal ovarian surface epithelia and inclusion cysts.

Unlike the epithelia outlining other organs, the ovarian surface epithelium is derived from the coelomic layer during development and is consisted of peritoneal mesothelial cells [35]. These uncommitted cells possess both mesenchymal and epithelial characteristics, adaptability suggested for post-ovulatory wound healing and tissue homeostasis [9, 35]. Many pioneered morphologic and profiling studies of OSE cells and ovarian cancer cells have found that malignant ovarian cancer cells express more epithelial markers than normal OSE cells [9, 10, 35-37]. E-cadherin expression was found in benign metaplastic cysts [38] and ectopic expression of E-cadherin in OSE caused mesenchymal-epithelial transition (MET) and the resulting cells formed tumors in immunodeficient mice [39, 40]. Moreover, elevated expression of E-cadherin in ovarian adenocarcinomas and the pre-malignant ovarian tissues was also identified in the laying hens, an animal model for spontaneous ovarian cancer derived from continuous ovulations [41, 42]. Together, all the reports indicate that E-cadherin-mediated metaplastic process precedes and is sufficient to cause ovarian pathogenesis [43]. In our study, knockdown of E-cadherin expression in OVCA432 ovarian cancer cells (Figure 5) showed that E-cadherin is important in inclusion cyst formation. It is intriguing that the MCAS cell line formed larger Matrigel spheroids after knockdown siRNA transfection. MCAS is the cancer cell line with the highest level of E-cadherin expression (Figure 4B), the marginal reduction in E-cadherin protein expression suggests that the regulation may be at the translational level, or other mechanisms such as predominant overexpression of miR200 and suppression of TGFβ signaling might be involved in spheroid formation in MCAS cancer cells (below). Nevertheless, knockdown of E-cadherin expression inhibited collective cell migration and induced single cell migration in both cell lines (Figure 5D), suggesting that the cellular level of E-cadherin also dictates how ovarian cancer cells migrate.

In addition to elevated E-cadherin expression, there might be other mechanisms such as integrin signaling that can carry out similar function. Baldwin *et al*. reported that CD151-α3β1 integrin complex was expressed in 58% of primary ovarian tumors and repressed slug-mediated EMT, although the integrin complex was suppressed in metastatic sites [44]. By employing cancer spheroid adhesion assay and a mouse model of ovarian cancer metastasis, Ip *et al.* showed that β1 integrin cooperated with P-cadherin to promote metastatic spread of ovarian spheroids onto the peritoneum via p70 S6 kinase activation [45]. Besides that, collective migration can also be caused by suppression of TGFβ function in ovarian cancer cells. Giampieri *et al.* reported that localized TGFβ signaling was activated in single mobile breast cancer cells, blockade of TGFβ signaling prevented cells from moving singly *in vivo* [46]. Our network analysis of microarray data has shown that miR200 family controls the switch between E-cadherin and TGFβ signaling (Figure 3). qRT-PCR has confirmed elevated expression of miR-200 family in the cancer 3D models, which was also consistent with the elevated expression of E-cadherin and downregulation of TGFβ2 in the cancer cells (Figure 4). The qRT-PCR data also showed dichotomous expression patterns of the miR200 family members. This might be related to their chromosomal localization in two separate miR200 clusters. The miR200a, miR200b, and miR429 are clustered in chromosome 1p36, and miR200c and miR141 are clustered in chromosome 12p13 [26]. The two clusters of miR200s might be under different regulations and potentially exhibit functional differences. It was also noted that MCAS cells showed predominant overexpression of miR200c, miR141 and miR205 when compared with other cell lines (Figure 4A), which might maintain the expression of E-cadherin and concomitant suppression of TGFβ signaling in a tightly regulated network that includes several negative feedback loops [47], a perturbation in the E-cadherin mRNA expression in the knockdown experiment might result in large MCAS spheroids in Matrigel (Figure 5B).

In conclusion, we have established sequential *in vitro* 3D culture models that mimic *in vivo* inclusion cyst formation and ovarian tumor invasion. Molecular profiling and functional analysis has suggested the significant role of E-cadherin of a miRNA-regulated EMT network in pre-malignant cyst structure formation and collective tumor invasion. The established 3D culture models will have utility in studying the contribution of this and other significant pathways in tumor initiation and progression.

## MATERIALS AND METHODS

### Antibodies

Here are the sources of our antibodies: E-cadherin, β-catenin, and GM130 (BD Biosciences, San Jose, CA); cleaved caspase-3 (Cell Signaling Technology, Beverly, MA); tenascin (Thermo Fisher Scientific, Pittsburgh, PA), laminin V (Millipore, Billerica, MA); collagen IV (Raybiotech, Norcross, GA); and β-actin antibody (Sigma-Aldrich, St. Louis, MO).

### Tissue specimens and cell lines

All patient-derived biologic specimens were collected and archived under protocols approved by the Human Subjects Committee of the Brigham and Women's Hospital, Boston, Massachusetts. Ovarian tissues were collected from women undergoing surgery at the Brigham and Women's Hospital for a diagnosis of primary ovarian cancer or from control subjects who underwent the procedure of hysterectomy or oophorectomy for benign diseases. All samples were collected with written informed consents from patients. The histopathological characteristics of the samples were provided by gynecologic pathologists and staged according to International Federation of Gynecology and Obstetrics (FIGO) system. The ovarian cancer cell lines have been described previously [48]. Normal human ovarian surface epithelial (OSE) primary cultures were collected by scraping the ovarian surface of the control subjects. Immortalized OSE cells were obtained using HPVE6E7 retroviral infection [49]. Tumor-associated fibroblasts (TAF) were derived from tumor tissue explants. All normal cells and cancer cells were established and grown in a mixture of medium 199 and MCDB105 medium (1:1) (Sigma, St. Louis, MO) supplemented with 10% fetal bovine serum (FBS, Invitrogen, Carlsbad, CA) as described previously [49]. TAFs were cultured in F12 medium (Invitrogen, Carlsbad, CA) supplemented with 10% FBS.

### Matrigel 3D cultures

Eight-well glass-chambered slides (BD Biosciences, San Jose, CA) were coated with growth factor-reduced Matrigel matrix without phenol red (BD Biosciences, San Jose, CA). Primary cultures of normal OSE and cancer cells were trypsinized and resuspended as single cells in complete medium with 10% FBS and 2% Matrigel. Fifty thousand single cells were seeded into each Matrigel-coated well. Complete medium with 2% Matrigel was replaced every 4 days. 3D structures would be formed after 4 to 7 days after seeding [24].

### Collagen 3D cultures

Spheroids formed in Matrigel were transferred to a microcentrifuge tube. Ultra-pure collagen-I solution (Sigma-Aldrich, St. Louis, MO) was neutralized and mixed with 5% FBS and complete medium and added to the spheroids. The mixture was then transferred quickly to the wells of a 24-well plate. Complete medium was added to cover the solidified matrix. The medium was replaced every 4 days and the structures were observed for about 20 days.

### Immunofluorescence microscopy

The 3D cultures were fixed in 4% paraformaldehyde (Sigma-Aldrich, St. Louis, MO) and permeabilized with PBS containing 0.5% Triton X-100 (Sigma-Aldrich, St. Louis, MO). After blocking with 10% FBS, primary antibodies were added and incubated at 24°C for 2 h. The 3D cultures were further incubated for 2 h with Alexa Fluor 647 conjugated secondary antibodies (Invitrogen, Carlsbad, CA) and Alexa Fluor 546-conjugated phalloidin (Invitrogen, Carlsbad, CA), and then post-fixed with 4% paraformaldehyde and counterstained with Sytox Green (Invitrogen, Carlsbad, CA). Images were captured using a Leica SP5 confocal microscope (Leica Microsystems, Bannockburn, IL) and analyzed by the Leica LAS AF software (Leica Microsystems, Bannockburn, IL). 0.1-μm interval z-section images were taken and 3D videos were constructed using Leica LAS AF software.

### Immunohistochemical staining of tissue samples

Archived formalin-fixed, paraffin-embedded specimens including five normal ovaries and fifteen ovarian tumor tissues were employed in the immunohistochemical staining. Seven-micron sections were cut from the paraffin-archived tissues, mounted on Superfrost Plus microscopic slides (Fisher Scientific, Pittsburgh, PA) and incubated at 50°C for 4 h. The sections were de-paraffinized in xylene and rehydrated with a descending series of ethanol. Antigen retrieval was performed in a pressure-cooker in antigen-unmasking solution (Vector Laboratories, Burlingame, CA). Endogenous peroxidases were blocked using 0.3% H_2_0_2_ in methanol for 20 min. The sections were then blocked with normal horse serum for 20 min and were subsequently incubated overnight with primary antibodies at 4°C. All primary antibodies were purchased from BD Biosciences (San Jose, CA) and were used in 1:100-200 dilutions. Alexa Fluor 647- or Alexa Fluor 546-conjugated secondary antibodies (Invitrogen, Carlsbad, CA) were diluted in 1:200. The tissue was counterstained with Sytox Green. Microscopic imaging was carried out as above.

### Gene expression profiling and network analysis

Total RNA was extracted using TRIzol reagent (Invitrogen, Carlsbad, CA) and submitted to the Center for Cancer Genome Discovery (CCGD) of Dana-Farber/Harvard Cancer Center for gene expression profiling using Affymetrix U133A 2.0 GeneChips (Affymetrix Inc., Santa Clara, CA) according to the instruction of manufacturer. Normalized gene expression data were uploaded to MetaCore™ Suite version 6.3™ (GeneGo Inc., St. Joseph, MI) for analysis. The “Compare Experiments” algorithm was used to identify the most significant pathways with statistics. The “Shortest paths network” algorithm was used to map potential signaling routes for the signaling network based on canonical pathways and expression data, with the most significant one highlighted in pink. The resulting network was converted into a map using MapEditor™ and uploaded back into MetaCore™ for concurrent data overlay of multiple experiments.

### Quantitative real-time reverse transcription PCR

TaqMan Reverse Transcription reagents (Applied Biosystems, Foster City, CA) were used for cDNA synthesis and TaqMan gene expression assays and TaqMan microRNA assays (Applied Biosystems, Foster City, CA) were used for qRT-PCR analysis in an Applied Biosystems 7300 Real Time PCR system (Applied Biosystems, Foster City, CA). Relative quantitation was calculated by 2^−ΔΔCt^ method and normalized to house-keeping gene cyclophilin A for mRNA expression, and RNU6B control for microRNA expression, respectively [50].

### Knockdown of E-cadherin in cancer cells

Mission^®^ siRNAs against human E-cadherin (SASI_Hs01_00086310 and SASI_Hs01_00086311) and Universal Negative Control siRNA were purchased from Sigma-Aldrich (St. Louis, MO). siRNA transfection was performed using Lipofectamine^®^ 2000 reagent (Invitrogen Corp. Carlsbad, CA). Knockdown of E-cadherin expression was confirmed by Western blot analysis.

### Statistical analysis

All calculations were performed with MINITAB statistical software (Minitab, State College, PA). ANOVA was used to compare the mean qRT-PCR scores between normal, benign and malignant samples to examine significant differences between groups. For other assays, significance of differences was determined using 2-tailed T-Test. A *P*-value of less than 0.05 was considered statistically significant for all tests.

## SUPPLEMENTARY FIGURES, TABLE AND VIDEOS


