# Supplementary material for: Loss of E-cadherin disrupts ovarian epithelial inclusion cyst formation and collective cell movement in ovarian cancer cells
Source: Oncotarget. 2015 Dec 13;7(4):4110–21. doi: 10.18632/oncotarget.6588 (PMC4826193; doi:10.18632/oncotarget.6588)

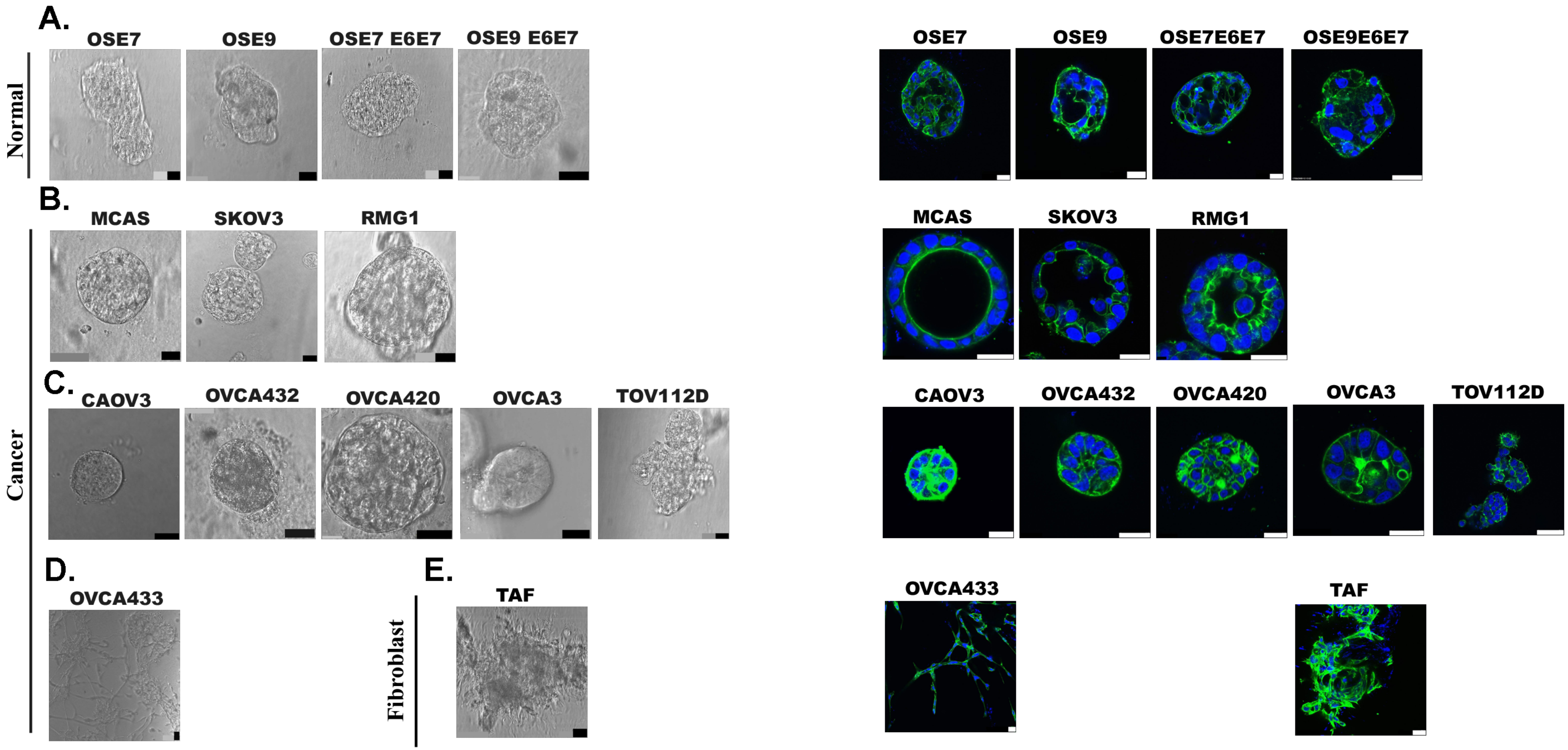

Figure S1. The morphologies of 3D Matrigel cultures of various ovarian cells.
Bright field images at 63X (left) and Confocal fluorescent images at 63X (right) of the ovarian cells in 3D Matrigel cultures. The ovarian cells are subdivided into: (A) Normal ovarian surface epithelial cells; (B) Ovarian cancer cell lines that form spheroids with lumen diameter > 20 μm; (C) Ovarian cancer cell lines that form spheroids without lumens or lumen diameter ≤ 20 μm; (D) OVCA433 cell line that forms branching 3D structures, and (E) Tumor-associated fibroblasts (TAF). The confocal images shown are overlaid images with cytoskeleton stained by phalloidin (green) and nuclei stained by Sytox Green (blue). The scale bar is 25 μm.

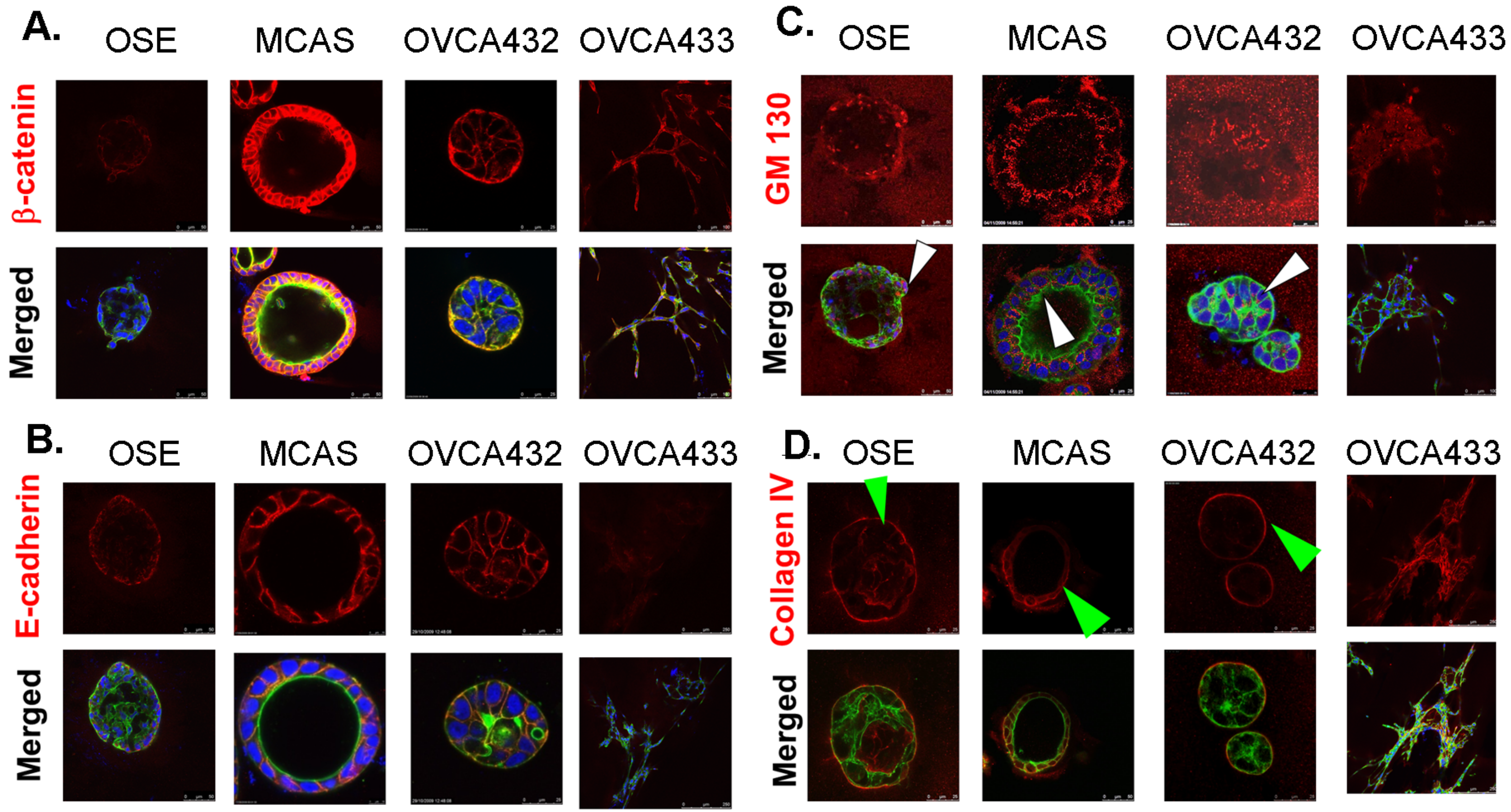

Figure S2. Laser scanning confocal images of (A) β-catenin, (B) E-cadherin, (C) GM130, and (D) Collagen IV in the 3D Matrigel cultures.

Confocal images at 63X of the 3D Matrigel cultures of different cell lines. The arrowheads in (C) and (D) indicate the positive apical GM130 staining, and basal collagen IV staining, respectively. The merged images are the overlaid images for the proteins of interest (red), F-actin (green) and nucleus (blue).

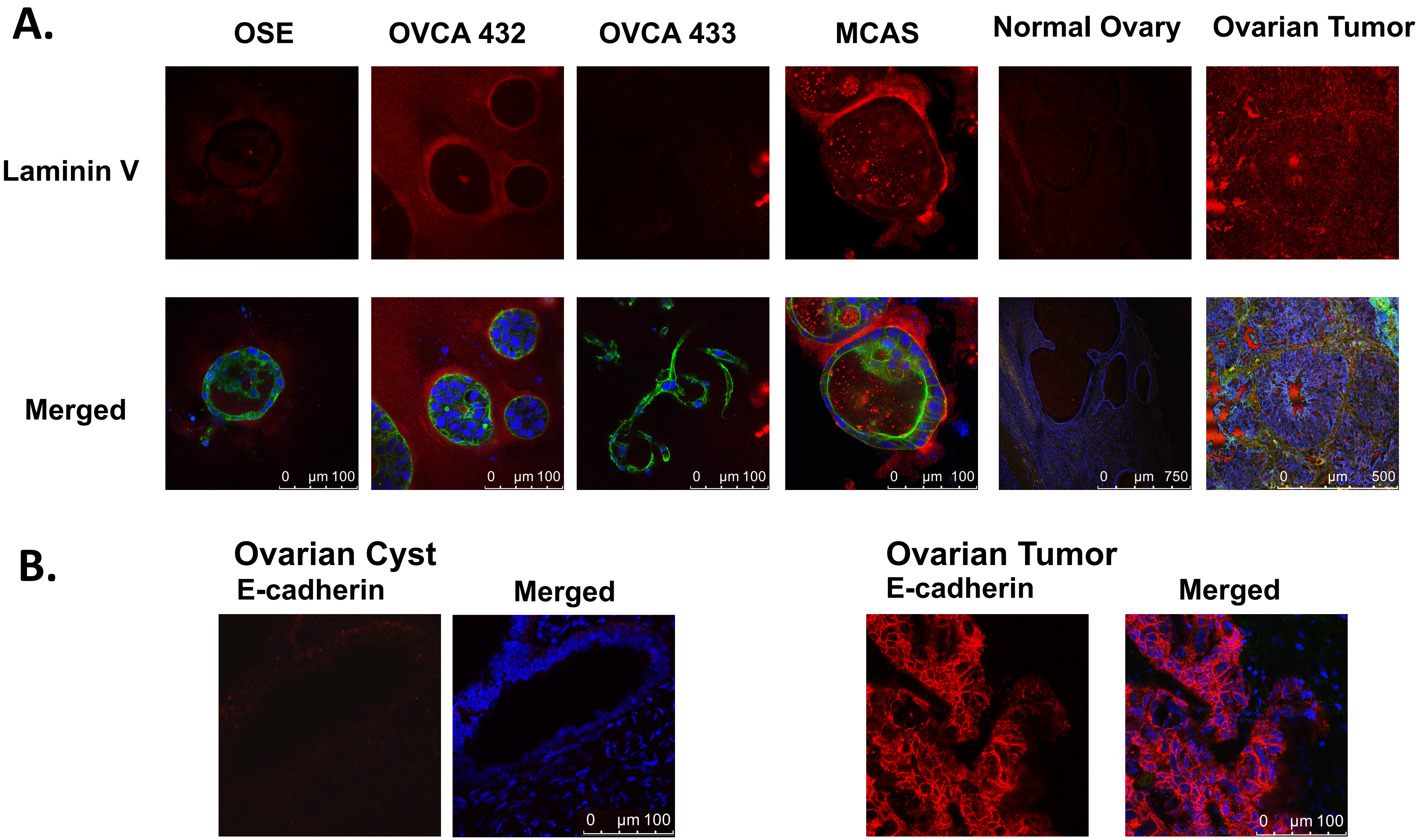

Figures S3. The expression patterns of (A) Laminin V and (B) E-cadherin in the 3D Matrigel cultures and clinical samples.

Confocal images at 63X of the 3D Matrigel cultures of different cell lines. The merged images in (A) are the overlaid images for Laminin V (red), F-actin (green) and nucleus (blue), whereas in (B), the merged images are the overlaid images for E-cadherin (red) and nucleus (blue).

Supplemental Table 1: Normalized expression data of the genes of the CDH1/TGFB pathway in cell lines under three growth conditions

Gene	Monolayer					Matrigel					Collagen				
	OSE9	OSE7	OVCA432	OVCA433	MCAS	OSE9	OSE7	OVCA432	OVCA433	MCAS	OSE9	OSE7	OVCA432	OVCA433	MCAS
CDH1	20.00	20.00	34.63	20.00	**148.37**	23.31	20.00	64.03	20.04	**292.39**	20.00	22.49	49.16	20.47	**129.86**
HOXA1	265.78	227.43	310.60	188.03	129.71	344.85	299.90	324.85	293.19	136.27	249.26	392.91	208.95	330.92	127.98
HOXA10	200.87	211.27	288.07	176.93	**1008.03**	371.96	290.01	372.67	211.72	**1811.70**	2130.38	2133.47	579.39	269.96	1580.96
HOXA11	32.52	27.12	23.15	24.80	**167.85**	108.72	36.76	27.52	30.12	94.21	**473.01**	**383.75**	43.36	31.38	**175.36**
HOXA2	408.12	374.21	179.41	193.84	130.43	319.99	526.16	92.77	236.53	145.22	345.64	276.22	96.35	187.26	138.58
HOXA4	**580.40**	**818.23**	51.35	51.08	63.25	**723.72**	**755.76**	44.09	83.96	105.36	**486.16**	**582.16**	90.98	101.13	82.17
HOXA5	313.10	**1816.29**	112.36	108.15	76.88	**636.62**	**1695.97**	157.27	172.23	115.06	**985.49**	**1361.90**	229.57	179.13	143.61
HOXA6	94.01	195.79	81.74	125.56	146.55	157.16	294.18	126.63	112.63	188.83	354.16	282.76	113.47	147.50	201.51
HOXA7	37.92	28.90	37.75	39.46	69.11	23.04	37.26	28.26	36.85	105.36	38.95	26.18	87.74	42.32	137.14
HOXA9	75.45	66.31	33.95	140.70	37.37	303.10	128.69	36.52	232.83	39.45	**1580.14**	**1769.46**	46.47	197.23	53.97
LEF1	20.72	23.53	20.00	42.99	**376.66**	77.45	27.38	27.24	33.18	164.19	55.96	179.56	259.55	53.52	202.07
MMP2	**2236.03**	**1265.53**	25.15	**1634.18**	25.59	**2858.16**	**3022.06**	33.34	**2913.73**	20.86	**2303.49**	**2201.92**	124.64	**2703.28**	22.92
SMAD2	917.81	1087.06	1716.48	845.40	684.12	1466.92	1548.06	1906.70	879.63	595.42	1352.56	1391.51	1672.85	740.00	717.10
SMAD3	1568.09	1358.30	1279.36	3172.87	2112.57	1236.30	1294.93	1810.22	3080.28	2164.63	1903.43	2196.24	774.80	3269.76	1691.57
SP1	188.13	174.90	298.71	143.89	223.42	148.32	162.20	423.53	104.98	471.94	137.22	202.03	161.96	103.70	299.53
TGFB1	367.29	242.30	205.84	876.43	373.85	983.36	450.65	768.86	1046.70	375.38	401.43	401.43	196.91	**1974.80**	290.41
TGFB2	3813.40	3254.10	51.87	1126.33	821.90	**1339.46**	**914.05**	43.67	122.79	**804.44**	**1689.48**	**1368.23**	169.23	52.18	131.30
TGFB3	68.04	282.45	66.42	81.27	76.35	78.31	485.66	74.67	66.43	166.97	164.91	76.97	133.14	51.53	155.52
TGFBR1	49.10	**206.99**	20.92	20.00	20.00	25.15	48.46	20.16	23.63	23.61	27.84	38.85	65.48	31.79	23.48
TGFBR2	5221.34	2974.40	1553.52	2553.25	6128.23	5283.86	3842.13	1477.14	4013.23	5087.77	4125.91	5621.22	835.66	2328.28	5049.79
ZEB1	3002.13	1590.27	1150.58	1717.71	134.87	**1845.63**	**1360.45**	418.71	**1638.85**	91.90	**1719.84**	**1143.87**	349.40	**1410.68**	40.22

Numeric characters of the cell lines under the same growth condition:

Bold > 4-fold of average of remaining cell lines

Shading (Fold-change relative to monolayer of the same cell line):

≤ 0.5 (blue) ≥ 2 (orange)

Video S1: Merged cross-sectional images of 3D Matrigel structure of OSE7 spheroids stained for E-cadherin (red), nuclei (blue), and F-actin cytoskeleton (green).

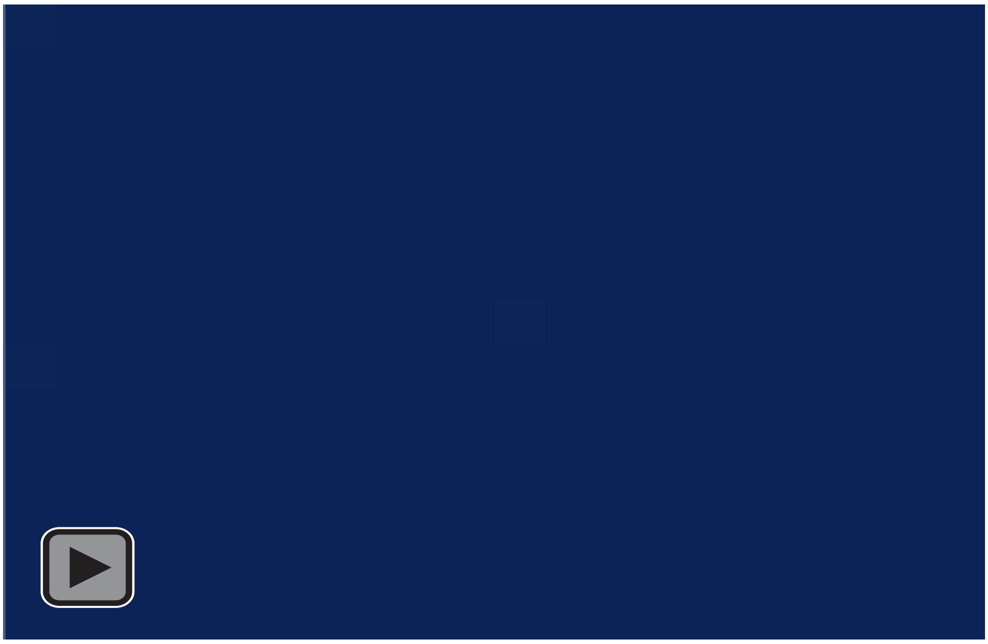

Video S2: Merged cross-sectional images of 3D Matrigel structure of spheroids formed by MCAS cancer cell line for E-cadherin (red), nuclei (blue), and F-actin cytoskeleton (green).

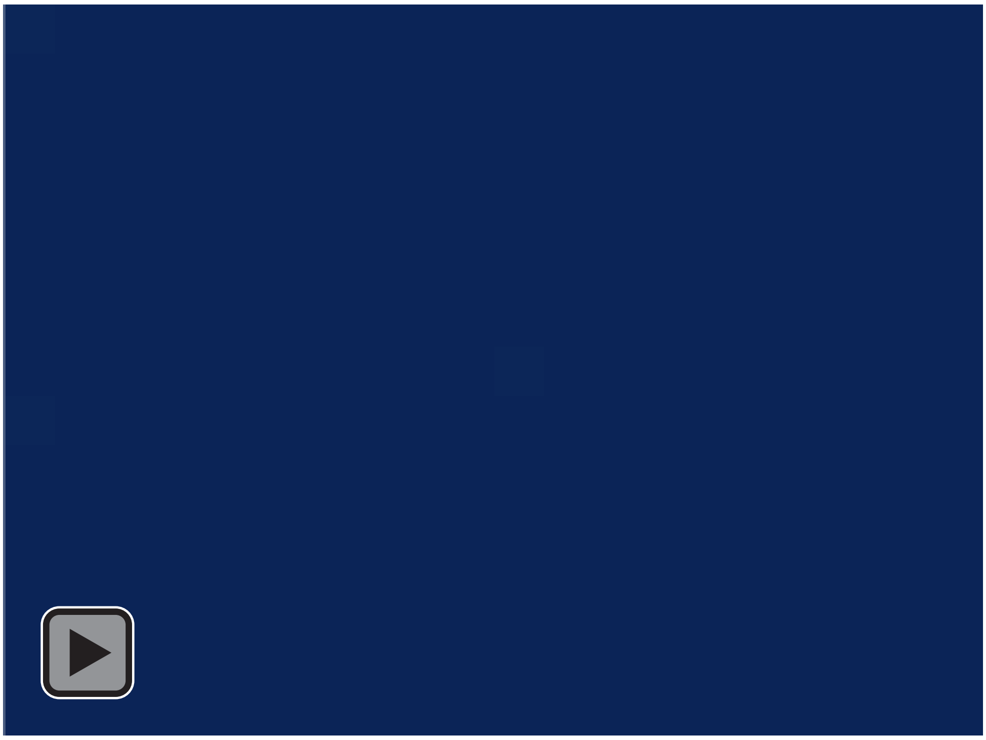

Video S3: Merged images of 3D Collagen I structure formed by MCAS cancer cell line for E-cadherin (red), nuclear DNA (blue), and cytoskeleton (green).

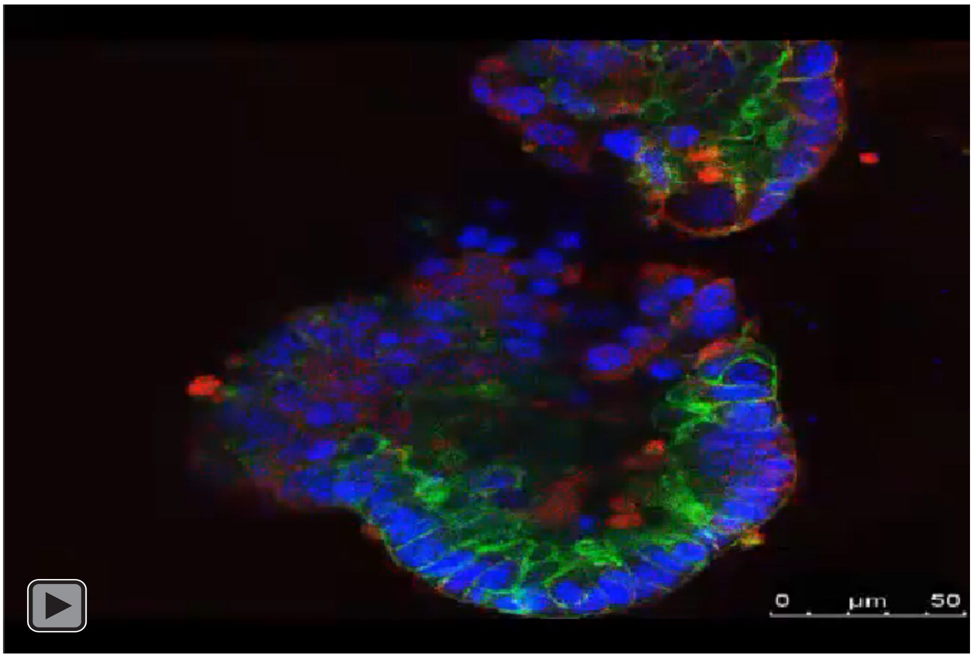

Video S4: Merged images of3D Matrigel structure formed by OVCA432 cancer cell line for E-cadherin (red), nuclear DNA (blue), and cytoskeleton (green).

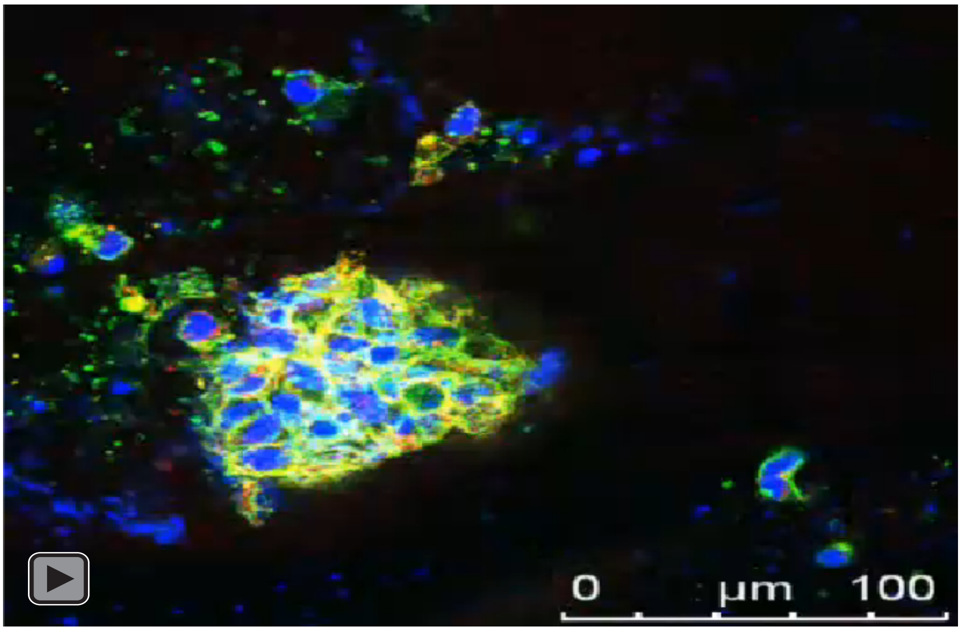

Supplement: Supplementary file 1 [file oncotarget-07-4110-s001.pdf]
